# Antioxidant and Antifatigue Activities of *Polygonatum Alte-lobatum* Hayata Rhizomes in Rats

**DOI:** 10.3390/nu6115327

**Published:** 2014-11-21

**Authors:** Chi-Ting Horng, Jon-Kway Huang, Hui-Yun Wang, Chi-Chang Huang, Fu-An Chen

**Affiliations:** 1 Department of Ophthalmology and Medical Education Center, Kaohsiung Armed Force General Hospital, Kaohsiung 80284, Taiwan; E-Mail: h560411@ms5.hinet.net; 2 Institute of Biochemistry and Biotechnology, Chung Shan Medical University and Chung Shan Medical University Hospital, Taichung City 40201, Taiwan; 3 Department of Pharmacy & Graduate Institute of Pharmaceutical Technology, Tajen University, Pingtung 90741, Taiwan; E-Mails: born.song@gmail.com (J.-K.H.); ppig8@yahoo.com.tw (H.-Y.W.); 4 Graduate Institute of Sports Science, National Taiwan Sport University, Taoyuan 33301, Taiwan

**Keywords:** *Polygonatum alte-lobatum* Hayata, antioxidant, anti-fatigue, biochemical parameters, exercised rats

## Abstract

*Polygonatum alte-lobatum* Hayata, a rhizomatous perennial herb, belongs to the Liliaceae family and is endemic to Taiwan. We investigated the antioxidant and anti-fatigue activities of *P. alte-lobatum* in exercised rats. Levels of polyphenols, flavonoids and polysaccharides and 2,2-diphenyl-1-picrylhydrazyl (DPPH) free-radical scavenging activity were measured in extracts of *P. alte-lobatum* (EPA). Sprague-Dawley rats were randomly divided into four groups for 8-week treatment with vehicle (control) and low-, medium-, and high-dose EPA (LEPA, MEPA, HEPA; 0, 75, 150, and 375 mg/kg/day, respectively). Exercise performance was evaluated by exhaustive treadmill exercise time and by changes in body composition and biochemical variables at the end of the experiment. EPA contained polyphenols, flavonoids and polysaccharides, with polysaccharide content at least 26 times greater than that of polyphenols and flavonoids. Trend analysis revealed that EPA dose-dependently scavenged DPPH free radicals. EPA treatment dose-dependently increased endurance running time to exhaustion and superoxide dismutase activity and total antioxidant ability of blood. EPA dose-dependently decreased serum urea nitrogen and malondialdehyde levels after exercise. Hepatic glycogen content, an important energy source for exercise, was significantly increased with EPA treatment. EPA could be a potential agent with an anti-fatigue pharmacological function.

## 1. Introduction

Fatigue is a common symptom in people with stress. Fatigue can be defined as a failure to maintain the required or expected force or power output [1]. In general, it can be divided into physical and mental fatigue [2]. With physical fatigue, exhaustion and free radical theories have attracted the most attention [3]. Delayed occurrence of fatigue and quick recovery are current foci of medical chemistry studies. Clinical drugs used to treat fatigue are limited, and potential alternatives from traditional medicine are worth investigating [4]. Recently, the use of traditional or herbal medicines has been increasing worldwide, so traditional or herbal supplements that can effectively ameliorate fatigue are important.

The functions of antioxidants in the maintenance of health and prevention of disorders or diseases have received great attention [5]. Antioxidant enzyme system helps protect against excessive or exhaustive exercise-induced oxidative damage and relates to physical status of athletes [6]. Antioxidant enzymes such as glutathione peroxidase, catalase, and superoxide dismutase (SOD) play important roles in preventing oxidative injury *in vivo* [7,8]. In high intensity or exhaustive exercise, overproduction of a large amount reactive oxygen species (ROS) could lead to oxidative stress and deleterious effects on tissue [9,10], and modulation of antioxidant enzymes activity may alleviate exercise-induced oxidative stress and body fatigue. Studies have suggested that enhanced antioxidant enzyme activity could prolong exercise performance and reduce physical fatigue. Therefore, supporting endogenous antioxidant systems with additional oral doses of antioxidants has been shown to prevent or reduce oxidative stress, decrease muscle damage, and improve exercise performance [11].

*Polygonatum alte-lobatum* Hayata belongs to the Liliaceae family and is a rhizomatous perennial herb that has been used as a tonic herbal drug [12]. The mature rhizomes are branched and have a yellow to green color. Recently, the plant is being used as a component of a herbal drink in Taiwan. Two new homologous series of 1,4-benzoquinones, 13 known compounds, and a novel alkaloid, polygonapholine, were isolated from rhizomes of *P. alte-lobatum* [12,13]. However, little information is available on the pharmacological activities of the plant, except for our previous study on its hypolipidemic activity [14]. Therefore, the primary purpose of our investigation was to evaluate the antioxidant and anti-fatigue activities of *P. alte-lobatum* in an exercised rat model.

## 2. Experimental Section

### 2.1. Materials

The rhizomes of *P. alte-lobatum* were collected from Nantou County, Taiwan. A voucher specimen (TU-LFA-100001) was placed in our research laboratory. Gallic acid, rutin, 1,1-diphenyl-2-pycrylhydrazyl (DPPH), Folin–Ciocalteu reagent, 2,2′-azinobis-(3-ethyl-benzothiazoline-6-sulphonic acid) (ABTS), galactose and 6-hydroxy-2,5,7,8-tetramethylchroman-2-carboxylic acid (Trolox) were from Sigma (St. Louis, MO, USA). Commercial diagnostic kits used to determine blood lactate and serum urea nitrogen (SUN) were from Roche Diagnostics (Basel, Switzerland). SOD and the total antioxidant capacity kit (ABTS scavenging assay) were from Randox Laboratories (Antrim, UK). All other chemicals were of analytical reagent grade.

Fresh rhizomes of *P. alte-lobatum* were sliced and air-dried in the shade. Rhizomes were pulverized and extracted with 20% ethanolic solution for 3 h in a reflux extraction apparatus (Angu, Kaoshiung, Taiwan), then filtered with filter paper and a funnel. The filtered liquid extract was further lyophilized to obtain EPA by freeze-drying (Panchun, Taipei, Taiwan). The obtained EPA was stored in an electronically operated dry cabinet (Komry, Taipei, Taiwan).

Total polyphenols in EPA were measured spectrophotometrically by use of Folin-Ciocalteu reagent based on a colorimetric oxidation/reduction reaction [15,16,17]. The amount of total polyphenols was expressed as microgram of gallic acid equivalent (GAE). Flavonoid content was based on the formation of chelatic colorimetrable compounds when reacted with aluminium chloride [18]. Briefly, 0.5 mL diluted EPA solution was prepared in 80% methanolic solution, then mixed with 0.15 mL of 10% aluminium chloride and 2 mL of 4% sodium hydroxide, then left to stand for 15 min at room temperature. The amount of flavonoids was calculated from the calibration curve of rutin standard solutions at 510 nm with use of a spectrophotometer (Hitachi, Tokyo, Japan) and expressed in milligrams per gram rutin equivalent (RE). The content of polysaccharides in EPA was determined by the phenol-sulfuric acid method [19,20]. Briefly, 1 mL EPA solution was mixed with 1 mL of 5% phenol solution and 5 mL concentrated sulfuric acids. The absorbance was measured at 490 nm (Hitachi, Tokyo, Japan) after shaking the mixtures in water bath at 30 °C for 30 min. The polysaccharide content was calculated from the calibration curve of galactose standard solutions. The antioxidant activity of EPA was evaluated with a DPPH free-radical scavenging assay as described [17,21].

### 2.2. Animals and Treatment

We used 24 male Sprague-Dawley (SD) rats from BioLASCO Taiwan maintained under standard laboratory conditions (12 h light/dark cycle, temperature 22 ± 2 °C). Rats (4 weeks old) were randomly divided into 4 groups for treatment: control (fed distilled water) and 75, 150 or 375 mg/kg EPA, for low-, medium- and high-dose EPA (LEPA, MEPA, HEPA, resepctively) with gavage feeding. Body weight and daily food intake was recorded within the 8-week experimental period. This study was approved by the appropriate animal care and use committees (approval #IACUC-26-100, Tajen University, Taiwan).

### 2.3. Treadmill Exercise Test

All rats were trained on a treadmill (rat/mouse treadmill T306, Singa, Taipei, Taiwan) for 12 days before an exhaustive exercise program described previously [17,22]. Motivation was provided by an electric shock zone at the rear of each compartment. Blood samples for serum urea nitrogen (SUN) and lactate acid levels were collected from an orbital puncture in rats before and after forced exercise for 20 min by using a microcapillary tube with rats anesthetized. On the day of the exhaustive exercise, rats were required to run to exhaustion on the treadmill at a final speed of 24 mph. The point of exhaustion was determined when the rat was unable to right itself when placed on its back.

### 2.4. Biochemical and Antioxidant Analyses

After exhaustive exercise, rats were killed by CO_2_ inhalation. Blood from hepatic veins was collected for evaluation of malondialdehyde (MDA), total antioxidant ability (ABTS scavenging assay) and SOD activity. Part of the liver in rats was used for hepatic glycogen testing [17,23]. Livers and kidneys of rats were cleaned, soaked in 10% neutral formalin solution, then embedded in paraffin wax and processed in a paraffin tissue-processing machine (Leica, Nussloch, Germany). Tissue was sectioned at 5 μm thick and stained with hematoxylin and eosin for histopathology assessment. Blood lactate level and SUN, SOD and total antioxidant activity were tested by use of commercial diagnostic kits, and MDA activity was measured as described [3,17].

### 2.5. Statistical Analysis

Data are expressed as mean ± SD. Statistical differences were analyzed by one-way ANOVA and the Cochran-Armitage test for trend analysis of the effect of EPA dose with use of SAS v 9.0 (SAS Inst., Cary, NC, USA), *p* < 0.05 was considered statistically significant.

## 3. Results and Discussion

### 3.1. Antioxidant Composition and Free Radical Scavenging Activity of EPA

We recovered 33.2% EPA from tissue for analysis of total polyphenols, flavonoids, and polysaccharides and DPPH free-radical scavenging activity. EPA polyphenol content was higher than that of flavonoid, and polysaccharide content was at least 26 times greater than that of polyphenol and flavonoid content (Table 1), so polysaccharides were abundant in EPA. The free-radical scavenging activity of EPA was evaluated by evaluating DPPH free-radical scavenging activity. The activity of DPPH inhibition was dose-dependent (*p* < 0.0001) (Table 2), with 50% inhibitory concentration (IC_50_) at about 9 μg/mL. This first report of the antioxidant activity of *P. alte-lobatum* suggests that the substantial antioxidant activity could be associated with composition of antioxidants, including total polyphenols, flavonoids and polysaccharides.

**Table 1:** Antioxidant composition of extract of *P. alte-lobatum* (EPA).

Ingredients	Amount
Polyphenols	14.29 ± 0.46 mg GAE/g
Flavonoids	9.97 ± 0.41 mg RE/g
Polysaccharides	365.46 ± 0.98 mg/g

Data are mean ± SD (*n* = 3). GAE: gallic acid equivalent; RE: rutin equivalent.

**Table 2:** 2,2-diphenyl-1-picrylhydrazyl (DPPH) free-radical scavenging activity of EPA.

EPA (μg/mL)	DPPH inhibition (%)
10	56.11 ± 1.51 ^d^
8	46.62 ± 0.57 ^c^
6	39.28 ± 1.49 ^b^
4	35.58 ± 0.16 ^b^
2	31.82 ± 3.03 ^a^
Trend analysis *P* value	**<0.0001**

Free-radical scavenging activity was evaluated as DPPH inhibition percentage based on reduced absorbance at 490 nm with doses of EPA for 30 min. Data are mean ± SD (*n* = 3). Different letters (a, b, c, d) indicate significant difference at *p* < 0.05 by the Cochran-Armitage test for dose-effect trend.

### 3.2. In Vivo Study of EPA Supplementation on Exercise Performance, Physical Fatigue and Antioxidant and Biochemical Variables Related to Fatigue in Rats

**Figure 1:**
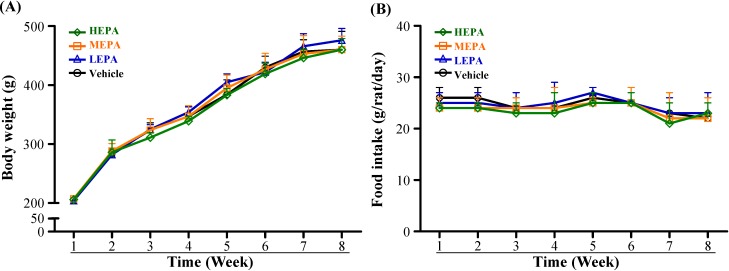
Effect of EPA on body weight (**A**) and daily food intake (**B**) of rats. Data are mean ± SD (*n* = 6). LEPA: low dose (75 mg/kg); MEPA: medium dose (150 mg/kg); HEPA: high dose (375 mg/kg).

The body weight of rats in the four groups increased from the beginning to the end of the experiment, but body weight and daily intake of food did not differ among groups (Figure 1). In a recent study, polysaccharides isolated from *Pleurotus tuber-regium* was shown to decrease body weights from week 4 and continued to week 8 in high-fat diet-induced increased body weight of diabetic rats. But body weights in polysaccharides supplemented groups approached to normal control animals [24]. Thus, EPA *per se* may have no effect on body weight and daily food intake of health-rats.

**Figure 2:**
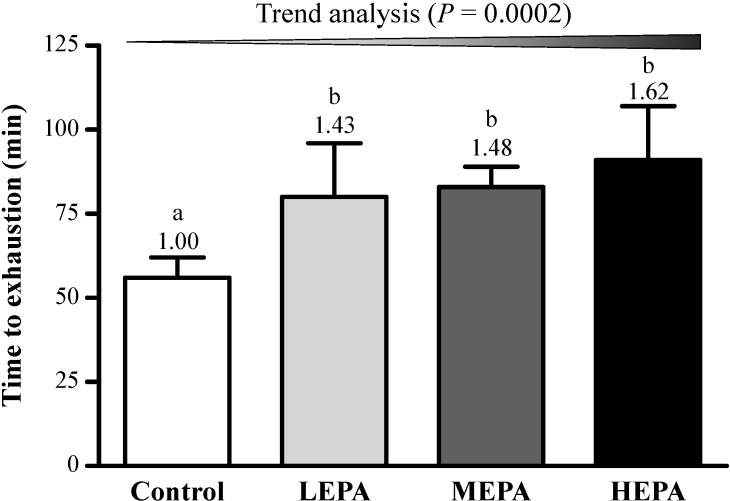
Effect of EPA on endurance time to exhaustion of rats. LEPA: low dose (75 mg/kg); MEPA: medium dose (150 mg/kg); HEPA: high dose (375 mg/kg). Data are mean ± SD (*n* = 6 rats per group). Different letters (a, b) indicate significant difference at *p* < 0.05 by the Cochran-Armitage test for dose-effect trend.

To evaluate the antifatigue effect of EPA, physical fatigue was measured by an exhaustive treadmill-running test. The test has been used broadly for measuring the antifatigue effects of herbal medicines [25]. Endurance with an exhaustive running test was higher for rats with than without EPA supplementation (Figure 2). For the trend analysis, endurance running time dose-dependently increased with EPA supplementation (*p* = 0.0002) and running time was longer with than without EPA by about 1.43-fold (*p* = 0.0057) to 1.62-fold (*p* = 0.0002). EPA was able to significantly extend the endurance time to exhaustion of rats, so it had antifatigue activity and could enhance exercise tolerance.

**Figure 3:**
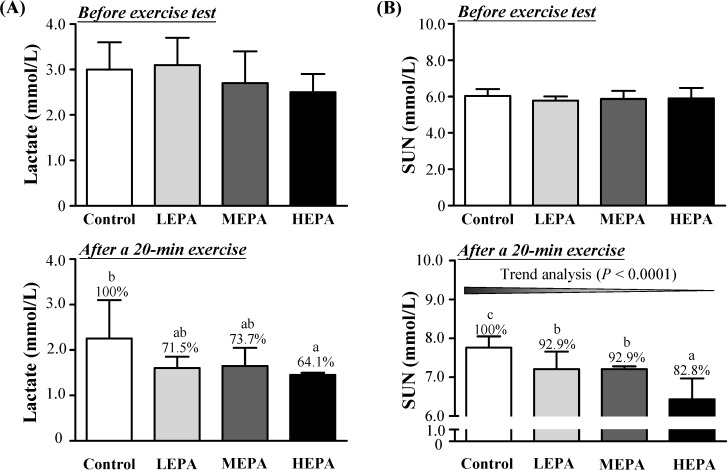
Effect of EPA on levels blood lactate (**A**) and serum urea nitrogen (SUN) (**B**) in rats before and after a single exercise challenge for 20 min. LEPA: low dose (75 mg/kg); MEPA: medium dose (150 mg/kg); HEPA: high dose (375 mg/kg). Data are mean ± SD (*n* = 6 rats per group). Different letters (a, b, c) indicate significant difference at *p* < 0.05 by the Cochran-Armitage test for dose-effect trend.

**Figure 4:**
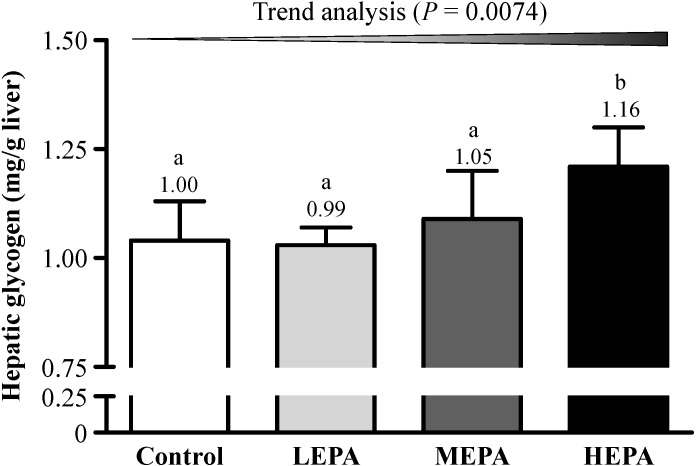
Effect of EPA on hepatic glycogen content in rats. LEPA: low dose (75 mg/kg); MEPA: medium dose (150 mg/kg); HEPA: high dose (375 mg/kg). Data are mean ± SD (*n* = 6 rats per group). Different letters (a, b) indicate significant difference at *p* < 0.05 by the Cochran-Armitage test for dose-effect trend.

To verify the effect of EPA on biochemical variables related to fatigue, we evaluated blood lactate, SUN, and hepatic glycogen levels. Before exercise, blood lactate and SUN levels did not differ between EPA doses and control treatment (Figure 3). After forced exercise for 20 min, blood lactate level was significantly lower with HEPA than control treatment (*p* < 0.05) (Figure 3A) and SUN content was lower for all EPA doses than controls (*p* < 0.05) (Figure 3B). Hepatic glycogen content was higher with HEPA than control treatment (*p* < 0.01) (Figure 4). Therefore, EPA has antifatigue activity by ameliorating altered biochemical variables related to fatigue in exercised rats. Blood lactate is the glycolysis product of carbohydrates after exercise challenge [26]. The accumulation of lactate can interfere with nerve impulses and muscle contraction, thus resulting in fatigue [27]. Ammonia is a metabolite of proteins and amino acids and is related to fatigue [28]. After a long time of exercise, SUN level will increase because of strong catabolic metabolism when the body cannot obtain enough energy by sugar and fat catabolic metabolism [29]. Therefore, decreased blood lactate and SUN levels is beneficial for relieving fatigue. Muscle glycogen content is exhausted after strenuous exercise, and energy is then derived from circulating glucose released from the liver via breakdown of glycogen [30]. Hepatic glycogen is an important variable related to fatigue. We found that EPA lowered blood lactate and SUN levels and increased hepatic glycogen level by reserving glycogen and/or reducing glycogen consumption, which can postpone physical fatigue. Many natural products and food factors are considered as supplements to improve fatigue symptoms. Recently, polysaccharides from medicinal plants have been studied to be a new sort of natural anti-fatigue agents [31,32,33]. In this study, we compared the fatigue-alleviating effects of 3 doses of EPA and vehicle on endurance in exercised rats. Ethanol extract of *P. alte-lobatum* dose-dependently enhanced exercise performance and reduced muscle fatigue physiological indexes, so *P. alte-lobatum* polysaccharides may have ergogenic and anti-fatigue functions.

To evaluate the antioxidant potential of EPA in rats, we evaluated total antioxidant (ABTS scavenging assay), MDA and SOD activity related to fatigue after exhaustive exercise. The ABTS free radical scavenging assay is based on the ability of antioxidants to scavenge the long-life radical cation (ABTS^+^) [34]. Total antioxidant ability was expressed as trolox-equivalent antioxidant capacity (TEAC). TEAC values were higher with all EPA doses than control treatment after exhaustive exercise (*p* < 0.05; Table 3), so EPA could increase the total antioxidant ability in exercise rats. In addition, SOD activity was higher with all EPA doses than control treatment (*p* < 0.05; Table 3). SOD is important in protecting against oxygen free radical damage that results in direct peroxidative damage to cellular components [35]. Exhaustive exercise can increase the degree of lipid peroxidation and reduce antioxidant activity [36]. We found that EPA could upregulate antioxidant enzyme activity to protect against oxidative stress induced by exhaustive exercise. MDA level was decreased in all EPA groups as compared with controls after exhaustive exercise (*p* < 0.01; Table 3). MDA, the end product of lipid peroxidation, is a good marker of free radical–mediated damage and oxidative stress. Endurance exercise can increase MDA activity [35,36]. Here we found that EPA had effective antioxidants preventing lipid peroxidation damage.

The anti-fatigue mechanism of *P. alte-lobatum* is still unclear. Different hypotheses for fatigue were proposed in light of its complexity and involvement of multiple factors [37]. Free radical theory has attracted much attention in antifatigue study. Free radical production during exercise contributes to fatigue, and antioxidant treatment might be a therapeutic approach [38]. We demonstrated that EPA contains antioxidant components such as polysaccharides, flavonoids and polyphenols and has free radical scavenging activity. The antioxidant components and free radical scavenging activity of EPA could be involved in ameliorating altered biochemical variables related to fatigue. We found no abnormal histopathologic changes in the liver and kidney of rats with EPA feeding for 8 weeks (data not shown), so EPA is safe as functional food.

**Table 3:** Effect of EPA on total antioxidant ability and superoxide dismutase (SOD) and malondialdehyde (MDA) activity in rats after exhaustive exercise.

Molecules	Control	LEPA	MEPA	HEPA	Trend Analysis *p* Value
TEAC (mg trolox/mg protein)	0.13 ± 0.05 ^a^	0.20 ± 0.04 ^b^	0.28 ± 0.08 ^c^	0.34 ± 0.03 ^c^	<0.0001
SOD (U/mg protein)	31.0 ± 13.7 ^a^	59.0 ± 12.7 ^b^	61.4 ± 25.5 ^b^	65.4 ± 16.5 ^b^	0.0005
MDA (nmol/mg protein)	0.78 ± 0.12 ^b^	0.40 ± 0.05 ^a^	0.41 ± 0.08 ^a^	0.39 ± 0.04 ^a^	0.0002

Data are mean ± SD (*n* = 6 rats per group). Different letters (a, b, c) indicate significant difference at *p* < 0.05 by the Cochran-Armitage test for dose-effect trend. TEAC: trolox equivalent antioxidant ability by the ABTS scavenging assay; LEPA: low dose (75 mg/kg); MEPA: medium dose (150 mg/kg); HEPA: high dose (375 mg/kg).

## 4. Conclusions

Taken together, we have demonstrated that treating rats with EPA could extend the endurance time to exhaustion and increase total antioxidant ability (TEAC values) and blood SOD activity, and decrease blood lactate and SUN and MDA levels. *P. alte-lobatum* seems to be a safe functional food that can ameliorate altered biochemical variables related to fatigue. EPA with anti-fatigue activity may be associated with its antioxidant components (polysaccharides, flavonoids and polyphenols) and free-radical scavenging activity. Further studies are needed to clarify the detailed mechanism(s) of the anti-fatigue properties of EPA.
